# Multi-capillary-column proton-transfer-reaction time-of-flight mass spectrometry^☆^

**DOI:** 10.1016/j.chroma.2013.09.072

**Published:** 2013-11-05

**Authors:** Veronika Ruzsanyi, Lukas Fischer, Jens Herbig, Clemes Ager, Anton Amann

**Affiliations:** a Department of Anesthesiology and Critical Care Medicine, Innsbruck Medical University, Anichstraße 35, A-6020 Innsbruck, Austria; b Breath Research Institute, Austrian Academy of Sciences, Austria; c Ionicon Analytik GmbH, Innsbruck, Eduard-Bodem-Gasse 3, Austria

**Keywords:** Proton transfer reaction time of flight mass spectrometry, PTR-TOFMS, Multi capillary column, Gas chromatographic separation, Volatile organic compounds, VOCs

## Abstract

Proton-transfer-reaction time-of-flight mass-spectrometry (PTR-TOFMS) exhibits high selectivity with a resolution of around 5000 *m*/*Δm*. While isobars can be separated with this resolution, discrimination of isomeric compounds is usually not possible. The coupling of a multi-capillary column (MCC) with a PTR-TOFMS overcomes these problems as demonstrated in this paper for the ketone isomers 3-heptanone and 2-methyl-3-hexanone and for different aldehydes. Moreover, fragmentation of compounds can be studied in detail which might even improve the identification. LODs for compounds tested are in the range of low ppb_v_ and peak positions of the respective separated substances show good repeatability (RSD of the peak positions <3.2%). Due to its special characteristics, such as isothermal operation, compact size, the MCC setup is suitable to be installed inside the instrument and the overall retention time for a complete spectrum is only a few minutes: this allows *near real-time* measurements in the optional MCC mode. In contrast to other methods that yield additional separation, such as the use of pre-cursor ions other than H_3_O^+^, this method yields additional information without increasing complexity.

## Highlights

• The coupling of PTR-TOFMS to a fast GC system using a multi-capillary column was successfully implemented.
• The built set-up enables the separation of structural isomers within seconds and does not hamper the normal operation of the PTR-TOFMS.
• The MCC setup is suitable to be installed inside the instrument and the overall retention time for a complete spectrum is only a few minutes.
• Besides, MCC-PTR-TOF allows a more accurate identification of compounds in complex mixtures.

## 1 Introduction

Proton-transfer-reaction mass-spectrometry (PTR-MS) has become a widely used technique in environmental science and biological research, permitting the monitoring of trace volatile organic compounds (VOCs) [1–3]. PTR-MS allows real-time analysis of breath, down to breath-to-breath resolution [4–10]. Replacing the quadrupole in a PTR-MS as a mass filter by Time-Of-Flight (TOF) mass separation opened new horizons to yield faster measurements, higher mass-range, and much more detailed information [11,12]. Quadrupole based PTR-MS instruments have unity mass resolution and compounds with the same nominal mass cannot be distinguished. PTR-TOFMS instruments possess a mass resolving power (*m*/*Δm*) of over 5000, where *m* is the respective mass (or *m*/*z*, more precisely) of the signal in the spectrum and *Δm* is the width (FWHM) of the peak. This high resolution represents a great step toward separation and identification of isobaric compounds, according to their exact mass.

However, the problem remains for compounds with the same molecular composition and thus the same exact mass. Employing different precursor ions for ionization such as O_2_^+^ or NO^+^ can be used for the differentiation of isomeric compounds [13]. While this can be of great use for target analysis of a certain limited number of compounds, this method usually fails for rich samples, where the comparison of the spectra for different pre-cursors becomes too complex.

Concerning fragmentation, the strength of dissociation depends on the difference in proton affinities between the analytes and the precursor ions (in the present study H_3_O^+^; 165.0 kcal mol^−1^) and the collision energy (E/N) in the reaction chamber. For humid sample typically a high E/N is chosen to suppress the formation clusters, which however, leads to more fragmentation and thus, to more complicated data.

Another method to gather additional information on sample composition is gas chromatography by adding another dimension of separation to the spectrum, based on the chemical properties of the compound. TOF-MS with electron impact (EI) ionization counts by now as standard GC detection technique.

The idea of coupling a PTR-MS to a commercial GC system has been implemented by several groups. As an example Lindinger et al. combined separation of VOCs by GC with parallel and simultaneous detection with PTR-MS and EI MS detection [14], respectively. However, in spite of the advantage of using a gas chromatographic separation regarding the increased selectivity of a GC-PTR-MS, this combination diminishes an important advantage of PTR instruments: their capability for real-time detection due to the long cycle period of GC measurements.

Sacrificing some of the temporal resolution of a regular GC, smaller size and shorter cycle times can be obtained by using a multi capillary column (MCC) instead. These columns have already successfully been implemented with other VOC gas analyzers [15,16]. Normally, a multi capillary column consists of around 1000 parallel capillaries bundled in a stainless steel tube. The inner surface of each capillary is covered by a film of a stationary liquid phase. Different models regarding the shape (straight or coiled) and different stationary phases are commercially available. The length of the column is normally between 40 and 250 mm permitting a smaller pressure difference across the column compared to packed and single capillary columns (e.g. with a length of 30 m). The bundle of capillaries enables a higher load capacity that can be used to get a higher sensitivity. The higher flow range of a MCC between 20 and 150 ml/min allows for isothermal separation and a simple and compact heating setup can be realized. Moreover, the high flow is favorable for the coupling to a PTR drift-tube, which requires a flow larger than 30 ml/min.

An MCC enables a fast gas chromatographic separation in near real-time and it is small enough to be installed inside a PTR-TOFMS instrument. The presented setup allows switching a PTR-TOFMS into an MCC-mode without adaptation to the instruments sampling procedure. From the construction point of view the use of an MCC is less expensive and less bulky than the coupling of a commercial GC system to a PTR-TOFMS.

We will present the employed setup and exemplify its capabilities. Two applications for measurements of complex VOC mixture such as human breath and human skin emission were selected and will be discussed. PTR-MS has already previously been applied in both fields [17–20]. With the present setup a more exact quantification of the single VOCs can be achieved, since addition of the signals from fragment ions arising from different compounds (e.g. in case of aldehydes emanated through skin) can be eliminated, and moreover, isomeric compounds can be separated.

## 2 Materials and methods

### 2.1 MCC-PTR-TOFMS

The following part describes the installation of a multi-capillary column in a PTR-TOFMS (PTR-TOF 8000, Ionicon Analytik, Innsbruck, Austria) [21]. Important instrumental parameters and reaction conditions of the PTR-TOFMS are listed in Table 1. The principles of operation of the instrument are described extensively elsewhere [11,22].

An important objective of the presented work was to implement a MCC for sample separation (1) without changing the normal operation parameters of the PTR-TOFMS and (2) while using the normal continuous sample gas inlet.

**Fig. 1:**
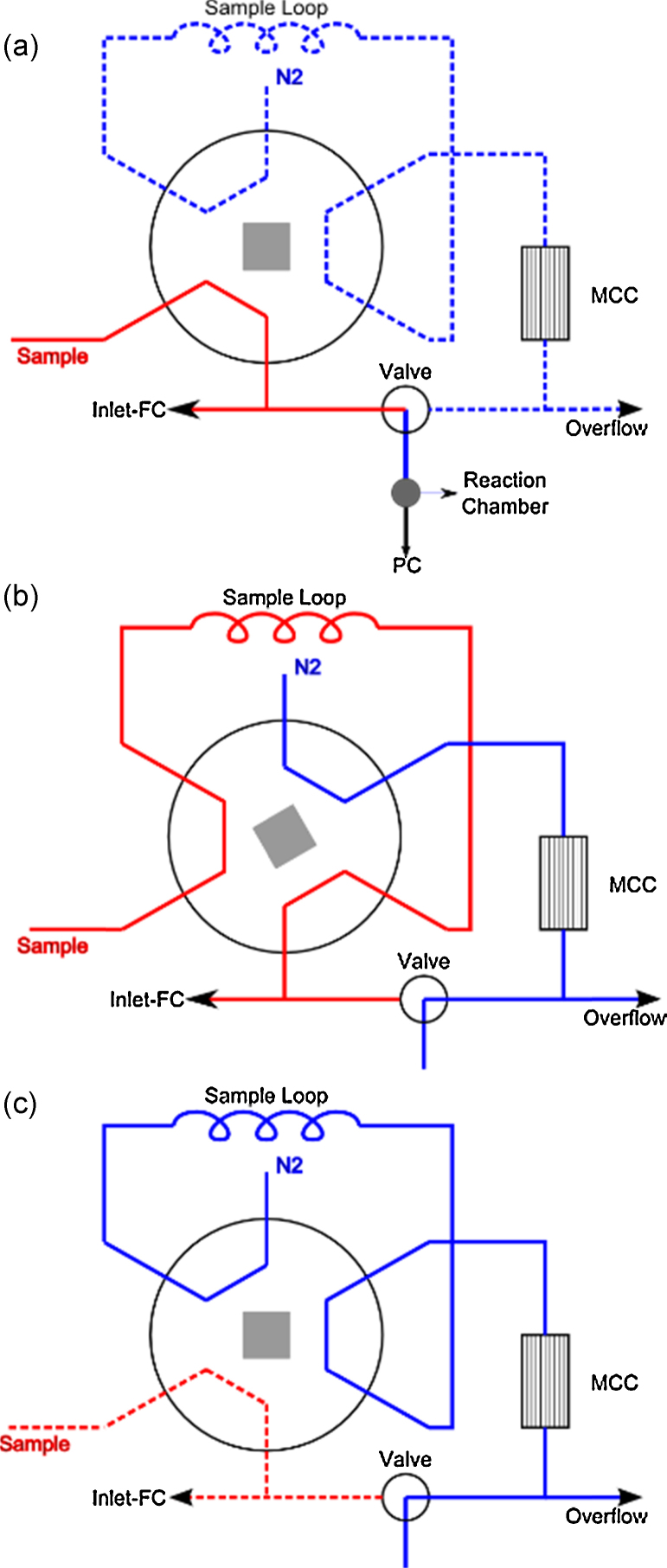
MCC sampling setup consisting of a valve and a 6-port valve (circle). The three main configurations are (a) real-time measurement, (b) filling of the sample loop, and (c) MCC measurement mode.

For operation of the PTR-TOFMS, we installed additional components in the PTR-TOFMS sample inlet system, as depicted in Fig. 1: the MCC, a 6-port-valve (ring), a sampling loop made of Teflon tubing (volume 5 ml), and an additional small 3-way-valve made of PEEK (3-way flipper valve Type 6650, Bürkert, Ingelfingen, Germany). The MCC (S2-40/OV-1/0.2, Multichrom, Ltd., Novosibirsk, Russia) used in our setup is 20 cm long, coated with 0.2 μm polydimethylsiloxane film as the stationary phase. In this prototype setup the 6-port valve is made of stainless steel, but should ideally be made of inert material, i.e. stainless-steel coated with Silconert2000^®^.

In order to control the MCC operating temperature, the multi-capillary column has been packed in an aluminum housing. Peltier elements between the aluminum housing and a heat sink allow the heating AND cooling of the column between 40 and 120 °C. This “mini-oven” is installed directly at the outside wall of the PTR-MS climate chamber. Therefore the sample gas connections to the MCC are still cold-spot free.

As displayed in Fig. 1, three different configurations of the valves are needed to operate the MCC-PTR-TOFMS:(a) *PTR-TOFMS mode*: This is the normal, real-time mode of the PTR-TOFMS only that in this setup the sample gas has to pass through the additional valves. The sample gas enters through the instruments normal sampling inlet and around 30 ml/min are drawn toward the pressure controller (PC) and the reaction chamber in total. To increase sample inlet flow, the inlet flow controller (“Inlet-FC”) can draw additionally between 0 and 1000 ml/min.

In this configuration the sample loop and the MCC are rinsed by a defined flow of N_2_ (nitrogen, purity 99.9999%), which is controlled by an additionally installed mass flow controller (not shown in Fig. 1, El_Flow, Bronkhorst High-Tech B.V., Ruurlo, The Netherlands).(b) *Sample-loop loading mode*: In this configuration the sample loop is flushed and is filled with sample gas after a few seconds. The Inlet FC can be used for faster filling of the sample loop.

Preferably, the small valve is already switched to direct clean N_2_ into the reaction chamber, to insure a low background.(c) *MCC mode*: In this mode the sample gas in the sampling loop is injected and pressed through the MCC by the flow of N_2_. The PTR-TOFMS now records the MCC chromatogram.

Following this protocol, the entire volume of the sampling loop is injected into the column, which defines the initial peak width. By switching back to configuration b) after a defined delay, the amount of sample gas injected and thus the initial peak width can be controlled. After injection the MCC measurement can be continued in configuration b) as well.

### 2.2 Preparation of test gases

3-Methylbutanal, hexanal, nonanal, octanal, 2-ethyl-1-hexanol, 3-methyl-2 hexanone, and decanal were purchased from Sigma–Aldrich (Steinheim, Germany), 3-heptanone from (Alpha Aesar GmbH Co KG (Karlsruhe, Germany).

For determination of the retention time and calibration of the compounds gaseous standards were prepared by evaporating liquid substances in glass bulbs. Each bulb (Supelco, Bellefonte, PA, USA) was cleaned with methanol (Sigma–Aldrich, Steinheim, Germany), dried at 85 °C for at least 20 h, purged with clean nitrogen for at least 20 min and subsequently evacuated using a vacuum pump (Vacuubrand, Wertheim, Germany) for 30 min. Liquid standards (0.5–1 μl) were injected through a septum, using a GC syringe. After the evaporation of standards, the glass bulb was filled with nitrogen of purity 6.0 in order to equalize the pressure (to ambient pressure). Then the appropriate volume [μl] of vapour mixture was transferred using a gas tight syringe (Hamilton, Bonaduz, Switzerland) into Tedlar^®^ bags (SKC 232 Series, Eighty Four, PA, USA, SKC 232 Series) previously filled with 2 l of nitrogen (purity 99.9999%).

### 2.3 Investigation for determination of flow and temperature characteristics

To determine effect of different temperatures on the peak characteristics the aldehyde mixture was measured in the same concentration (138 ppb for 3-methylbutanal, 121 ppb for hexanal, 90 ppb for octanal, 81 ppb for nonanal, 74 ppb for decanal) using 50 ml/min flow rate by different temperatures (isothermal during the run), namely 40, 50, 60 and 80 °C. To investigate the influence of different carrier gas flows on the separation the same mixture was measured at 50 °C by applying flow rates of 10, 20, 50, 70, 100 and 140 ml/min.

### 2.4 Procedures for monitoring of aldehydes and ketones in human samples

Two exemplary applications were selected for demonstrating the MCC-PTR-TOFMS for monitoring trace levels of VOCs: skin emanation and human breath analysis. The here tested volunteer/patient gave their written informed consent. The skin and breath measurements were approved by the ethics committee of Innsbruck Medical University.

#### 2.4.1 Skin emanation

For sampling of VOCs emanated through skin, the same setup was used as described in [23]. The stainless steel pan was fixed onto the navel area of the volunteer during the measurements. The outlet of the metal jar was connected directly with the PTR-TOFMS inlet using 1/8″ Teflon tubing. The inlet of the jar was coupled to the gas supply delivering nitrogen (99.9999% purity) with a flow rate of 20 ml/min.

A T-piece between the gas supply and the jar ensured the overflow of nitrogen according to the flow requirement of the PTR-TOF controlled by the temperature of the drift tube with the reason that no room air attains into the system. For sampling the 6-port valve was switched to position b) as displayed in Fig. 1. For accumulation of the VOCs in the pan, the inlet flow of the PTR-TOF MS, which sucks the sample from the jar through the sampling loop in this valve position was set to zero for 5 min. Then the loop was filled with sample gas by setting 20 ml/min sample flow and after 3 s the sample from the loop was injected into the MCC by switching to position “c” (MCC mode displayed on Fig. 1).

#### 2.4.2 Breath gas sample

Breath samples were collected in a CO_2_-controlled manner in Tedlar^®^ bags at the Department of Neurology of the Innsbruck University Hospital as described in [24]. Samples were measured within 30 min after collection. Within this time window we did not observed any loss of the compound by testing the prepared gas standards as also stated in [25] for other ketones. The Tedlar^®^ bag was connected directly to the sample loop inlet of the PTR-TOFMS.

#### 2.4.3 Analysis parameter

Table 1 summarizes the analysis parameter for detection of breath and skin volatiles and related gas standards produced.

## 3 Data evaluation

The typical PTR-TOFMS measurement and data evaluation has been described extensively in the literature e.g. recently by Müller et al. [26]. Here we followed the standard strategy: The TOF produces a full scan every 40 μs.

25,000 of these spectra are integrated to increase the signal-to-noise ratio and reduce the amount of data. Consequently, we record one integrated spectrum every 1 s for duration of 4 min. The TOF data acquisition software TofDaq Recorder (vers. 1.2.93, TOFWERK AG, Thun, Switzerland) exports this data in HDF5 format.

For processing of the spectra a custom made software was created to perform the following steps:

a) re-calibration of the mass-scale for each spectrum (using H_3_O^+^, H_2_OH_3_O^+^, NO^+^).
b) extraction of the signal for the compounds of interest by integrating the counts around the exact mass. For the aldehydes the most abundant ions 69 and 83 were selected and integrated using the borders 68.72–69.40, and 82.71–83.43, respectively.
  For the ketones, the main peak at 115 was chosen with the range of 114.86–115.33 for integration. The ranges were deliberately selected wide to accustom for possible fluctuations in the mass calibration.
c) the resulting time traces are provided in excel tables for further processing.

The time profiles were then visualized in Excel and manually integrated by selecting a retention time range and summing the areas (signal × duration). To estimate the peak retention time and full width at half maximum (FWHM) we fitted the peak shape to a normal distribution. For the retention time this is equivalent to calculate the center of gravity of previously calculated area. To obtain FWHM the standard deviation of the fitted distribution needs to be multiplied by the factor $2\sqrt{2\;\ln\;2}$ which amounts to approximately 2.355.

The distribution of calibration curve parameters were obtained through bootstrapping simulation as this is a novel method and bootstrapping does not require assumptions about the nature and range of measurement noise.

To determine detection and quantification limits we used the defining relations as stated in [27], (Section 2.3.1), the reported critical values are then: detection decision (LC, false positive risk <5%), detection limit (LOD, false negative risk <5%) and quantification limit (LOQ, RSD of quantification <10%).

## 4 Results and discussion

We present two applications of MCC in combination with PTR-TOFMS: detecting trace quantities of VOCs emanating through skin and from breath. A fast separation can be useful in numerous cases, such as the pre-separation of the sample gas humidity, to avoid the influence of the humidity on the quantification. In the present example we made use of a more exact quantification by preventing the overlap of compounds and of fragment ions.

### 4.1 Detection of aldehydes in skin emanation

Aldehydes are characteristic VOCs for skin emission [28]. Since it is well known that most aldehydes fragment after protonation, we studied the fragmentation pattern of the most abundant aldehydes emanation through human skin using PTR-TOFMS. In general, compounds, which contain a hydroxyl group (alcohols) and in some cases also a carbonyl group (aldehydes containing three and more carbon atoms) show loss of neutral water from the protonated molecule as the first stage of fragmentation [29]. The relative abundance to the most dominant signal for the selected aldehydes is displayed in Table 2.

The investigated aldehydes exhibit similar fragmentation pattern. For octanal, nonanal and 3-methylbutanal the most abundant *m*/*z* at 69.07 can be observed in the PTR-TOFMS spectrum. Additionally, decanal exhibits this ion with 29% relative abundance. Furthermore, the ion at *m*/*z* 83.07 appears with the highest abundance for hexanal and decanal simultaneously and with 58% rel. abundance in the spectrum of nonanal. Thus, these fragment ions could not be used for quantification if all these compounds are in the sample gas. However, the parent ion can be detected for all of the chosen substances (3-methylbutanal: 87.08 (21%), hexanal: 101.09 (12%), octanal: 129.15 (24%), nonanal: 143.04 (55%), decanal: 157.16 (6%)), which could be utilized for quantification by correcting for the fragmentation ratio. Unfortunately, change in the gas matrix, such as the humidity level, might influence the fragmentation; therefore the exact quantification of one single aldehyde can become difficult for samples where the humidity content might change, such as skin emanations.

Therefore we applied the MCC for fast separation of the aldehydes within the time range of seconds, which enables stable retention times with RSD smaller than 3.2% as displayed in Table 3. The standard deviations are in the range of 0.3–0.6 s for the compounds and they seem to not differ much. The small variation of RSDs can be explained through the division with the mean retention times between 10 and 160 s. The most abundant ion (see Table 2) was selected for every aldehyde for determination of parameters for separation and quantification.

Regarding the detection limits we computed higher values (between 1.73 ppb_v_ for 3-methyl-2-hexanone and 13 ppb_v_ for decanal) compared to the numbers known from the literature for PTR-TOF MS, which are in the low ppt_v_-range in the majority of cases. The reason for this lays in the dilution effect of the carrier gas by injection of the sample volume of 5 ml into the column. In the normal PTR-mode a continuous sample flow is introduced over a longer time enabling lower LODs.

**Fig. 2:**
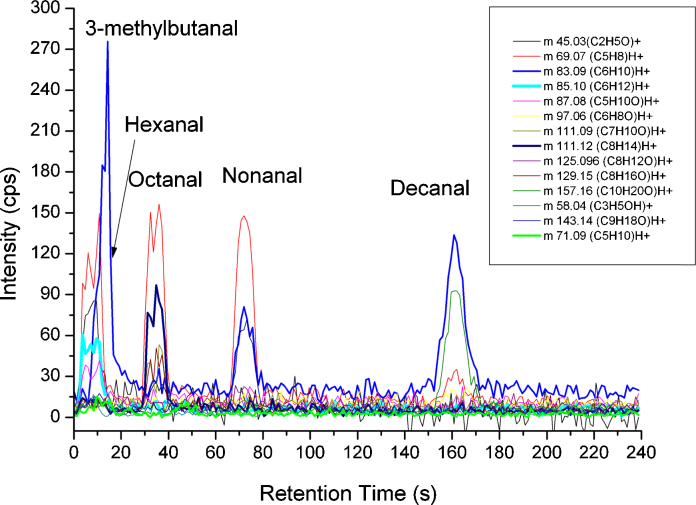
Chromatogram of aldehyde mixture measured with MCC-PTR-TOF.

Fig. 2 displays the chromatogram of the above specified aldehydes (3-methylbutanal: 16.0 ppb_v_, hexanal 16.8 ppb_v_, octanal 17.2 ppb_v_, nonanal 16.0 ppb_v_, decanal 16.4 ppb_v_ in the gas mixture). The chromatogram shows a clear separation of the compounds with exception of hexanal and 3-methylbutanal, which overlap, but fortunately do not exhibit the same fragment ions.

#### 4.1.1 Flow characteristics

**Fig. 3:**
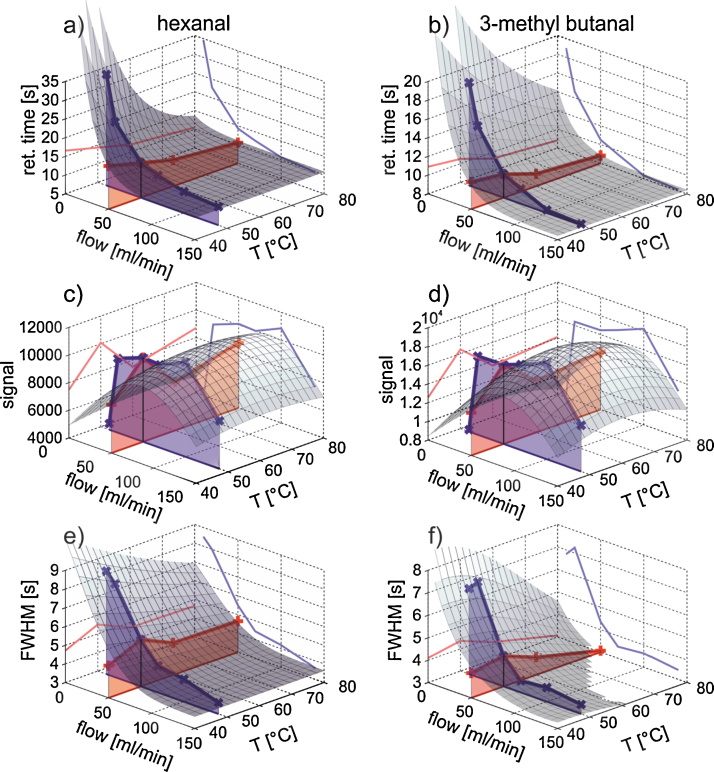
Influence of temperature and flow on peak retention time, peak signal and peak width with hinted surface of relation. (a) Retention time for hexanal; (b) retention time for 3-methyl butanal; (c) peak signal for hexanal; (d) peak signal for 3-methyl butanal; (e) FWHM for hexanal; (f) peak FWHM for 3-methyl butanal.

We examined the signal characteristics at different temperatures and flows by holding the remaining parameters constant as described in Section 2.3. As expected there is a much stronger influence on the retention times and FWHM when changing the flow rate in the range of 20–70 ml/min. The variation of the temperature does not affect strongly the FWHM and peak positions in the range examined (the maximal possible temperature was 80 °C) as can be seen on Fig. 3 pictured on the example of hexanal and 3-methylbutanal.

Regarding the signal height, we can find a maximum range between 40 and 100 ml/min for both compounds. Here also, the changing of the temperature does not impact the peak intensities significantly.

#### 4.1.2 Skin emanation sample

**Fig. 4:**
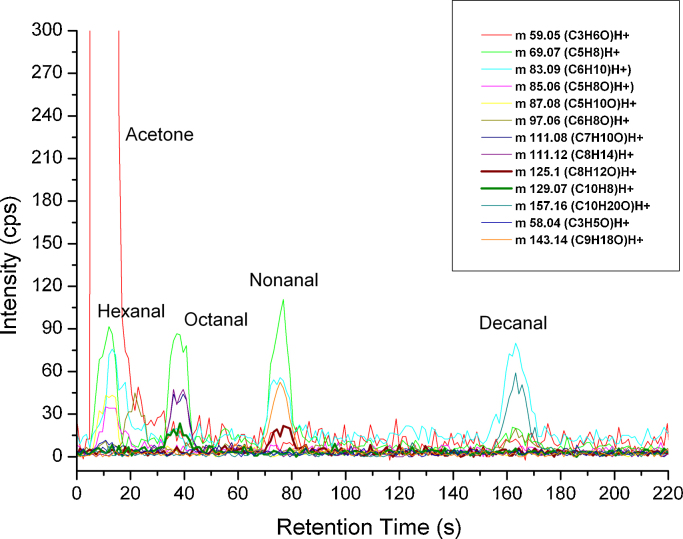
Chromatogram of skin emanations containing acetone, hexanal, octanal, nonanal and decanal detected by MCC-PTR-TOFMS.

In Fig. 4 a skin emanation sample of a volunteer can be seen. Besides decanal, nonanal, octanal, and hexanal high concentrations of acetone could be detected. The release of the latter by skin was confirmed by Turner at al. [30]. The calculated concentration for decanal is 29.9 ppb_v_ for nonanal 14.4 ppb_v_, for octanal 8.5 ppb_v_, and 4.9 ppbv hexanal using the most abundant ions for every compound according to Table 2.

### 4.2 Separation of ketone isomers

Ketones show a very low fragmentation in the chemical ionization process, thus mainly their protonated parent ion will be observed. Therefore, the separation and quantification of these compounds is uncomplicated in the majority of cases with exception of isomers having the same molecular weight.

To demonstrate the separation capability of MCC we selected two ketones, 3-heptanone, which is a metabolite of 2-propylpentanoic acid (valproic acid) and 3-methyl-hexanone as a potential indoor air contaminant. 2-Propylpentanoic acid is used as antiepileptic drug primarily in the treatment of epilepsy, and major depressive disorder.

**Fig. 5:**
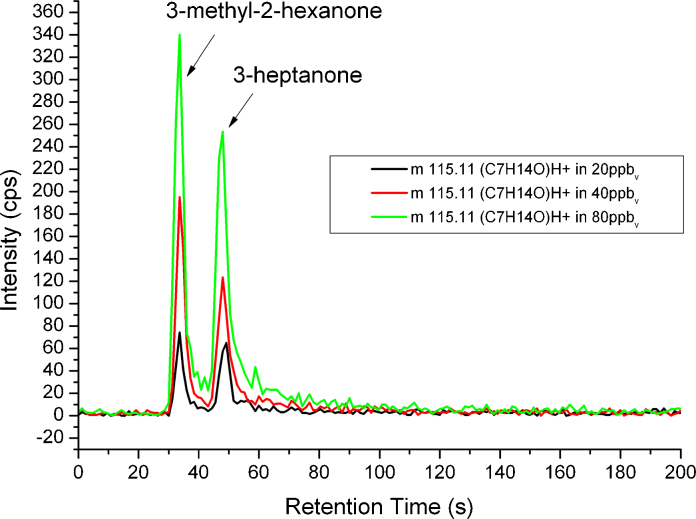
Separation of constitutional isomers, 3-heptanone and 3-methyl-2-hexanone.

Fig. 5 displays the detected signal for *m*/*z* 115.11 in prepared test gas mixture for 20, 40 and 80 ppb_v_ (for each substance). Two separate peaks can be observed from the same ion at retention times of 34.0 s and 48.5 s belonging to 3-methyl-2-hexanone and 3-heptanone, respectively. Within 60 s both compounds are eluted from the column. Table 3 summarizes parameters of the chromatographic separation and calibrations.

**Fig. 6:**
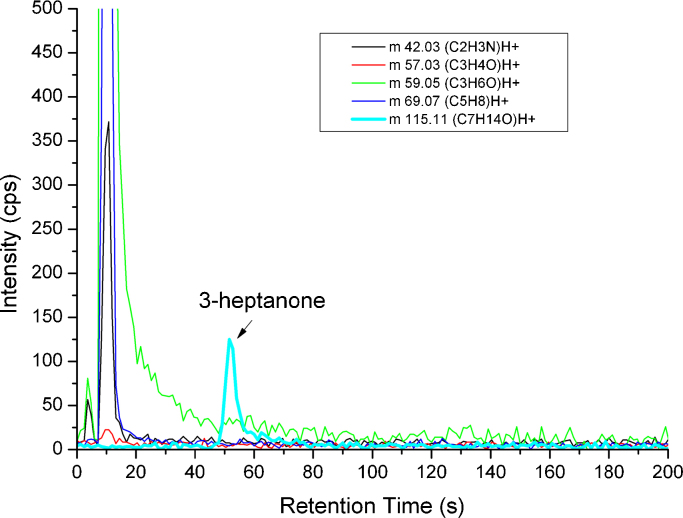
Breath sample of a patient under valproic acid therapy measured by MCC-PTR-TOFMS.

In the exhaled breath sample of the examined patient under 2-propylpentanoic acid therapy 3-heptanone could be detected exclusively on *m*/*z* 115.11 at 49.0 s as shown in Fig. 6. The concentration was calculated as 49 ppb_v_. Besides, the patient's breath sample contained several other compounds such as isoprene at *m*/*z* 69.07, acetone at 59.05, acetonitrile at 42.03. These last compounds eluted simultaneously using the given chromatographic settings, since the task of the current study was to rapidly separate isomers of *m*/*z* 115.11. However, with change of the parameters, like reduction of the flow rate, it might be possible to separate these compounds with the resulting concomitantly longer analysis run.

## 5 Conclusions

We have successfully implemented the coupling of a MCC column with a PTR-TOFMS system. The characteristics of the fast GC separation, i.e. comparatively fast flow, high sample volume, small dimensions, present an ideal combination with a PTR-TOFMS. This provides an additional dimension to the data, which adds highly valuable information with only a moderate sacrifice to the real-time capability of the instrument. The amount of information provided a PTR-TOFMS can already be challenging to process. However, adding the additional dimension does not necessarily complicate the process: The time-of-flight and retention-time dimension do not influence each other, i.e. the exact mass of the compound is the same with or without the MCC. As a result, the two data analyses can be regarded as separate problems. Moreover, the simplest way to extract concentration data from a TOF spectrum is to integrate the signal over a certain mass range around the exact mass of an ionized compound. This simple process only fails when the signals of several compounds overlap, which can be improved by the MCC pre-separation.

We have demonstrated the separation of constitutional isomers (3-methyl-2-hexanone and 3-heptanone) in a PTR-TOFMS spectrum. In addition, five different aldehydes forming common fragment ions are separated with the MCC giving the possibility of a simplified and more exact quantification. The functionality of the newly constructed set-up was proven on real samples containing VOCs in the low ppb_v_-range such as skin emanation and human breath.

In the presented setup, all necessary parts have been installed inside the PTR-TOFMS instrument without affecting the normal mode of real-time analysis. It is therefore possible for the user to extract additional information by adding MCC-PTR-TOFMS spectra to their experiments without adapting their normal PTR-TOFMS sampling procedure. In the future MCC separation might also be used to eliminate compounds with high concentration that would hinder the measurement, e.g. different solvents; among others hexane, which enables the simple preparation of test gases from weakly water-soluble substances such as aldehydes. Moreover in other biological applications, such as head-space analysis of alcoholic beverages ethanol could be eliminated, or the humidity in case of breath analysis.

## Figures and Tables

**Table 1:** Analysis parameters for VOCs detection using MCC-PTR-TOF.

Parameter	Analysis of aldehydes/skin sample	Analysis of ketone isomers/breath sample
Drift inlet pressure	2.14 mbar	
Drift inlet temperature	80 °C	
Transfer line temperature	120 °C	
Drift Field (and resulting E/N)	600 V (140 Td)	
Inlet flow for rinsing the loop	20 ml/min	
Pressure TOF lens	6.4 × 10^−6^ mbar	
MCC temperature	50 °C	40 °C
Carrier gas flow	50 ml/min	20 ml/min
TOF extraction frequency	25 kHz	
Number of scans per analysis	240	
Analysis time	4 min	
Mass range	*m*/*z* 0.0–508.47	

**Table 2:** The relative abundance normalized to the most prominent signal for 3-methylbutanal, hexanal, octanal, nonanal and decanal. Protonated parent ions are marked with bold letters.

Compound	*m*/*z* of the protonated parent and fragment ions and their relative abundances				
3-Methylbutanal	69.07 (100%)	45.03 (43%)	**87.08 (21%)**		
Hexanal	83.07 (100%)	55.07 (49%)	**101.09 (12%)**	58.02 (4%)	
Octanal	69.07 (100%)	111.09 (59%)	**129.15 (24%)**	85.08 (4%)	
Nonanal	69.07 (100%)	83.07 (58%)	**143.04 (55%)**	85.08 (34%)	125.09 (24%)
Decanal	83.07 (100%)	69.07 (27%)	97.10 (18%)	**157.16 (6%)**	

**Table 3:** Retention times (*R*_*t*_), RSDs (%), detection decision (DC), LODs, LOQs and correlation coefficients (*R*^2^) and the uncertainty of the slopes obtained for the aldehydes and ketones under study using MCC-PTR-TOF.

Compound	CAS	*R*_*t*_ (s)	RSD (%)	*R*^2^ 50%	Slope 50% (20–75%)	DC (ppb)	LOD (ppb)	LOQ (ppb)
3-Methylbutanal	590-86-3	10.04	3.2	0.98	102.23 (100.39–104.47)	2.94	5.85	14.57
Hexanal	66-25-1	13.93	2.0	0.97	66.97 (65.66–68.83)	3.15	6.23	15.02
Octanal	124-13-0	35.22	1.0	0.97	46.38 (45.65–47.29)	2.55	5.05	12.28
Nonanal	124-19-6	69.68	0.5	0.94	23.01 (22.41–23.64)	3.45	6.76	16.18
Decanal	112-31-2	161.12	0.4	0.85	16.04 (15.82–17.11)	6.69	13.04	29.84
3-Methyl-2-hexanone	2550-21-2	33.4	1.0	1.00	92.32 (91.27–93.29)	0.88	1.73	4.42
3-Heptanone	106-35-4	46.4	0.6	0.99	125.65 (124.09–126.91)	0.61	1.20	3.27
